# Socioeconomic, ethnic and geographical disparities in revascularization rates for peripheral arterial disease in England

**DOI:** 10.1093/bjsopen/zrag081

**Published:** 2026-08-05

**Authors:** Ravi Maheswaran, Thaison Tong, Jonathan Michaels, Paul Brindley, Stephen Walters, Shah Nawaz

**Affiliations:** School of Medicine and Population Health, University of Sheffield, Sheffield, UK; School of Medicine and Population Health, University of Sheffield, Sheffield, UK; School of Medicine and Population Health, University of Sheffield, Sheffield, UK; School of Architecture and Landscape, University of Sheffield, Sheffield, UK; School of Medicine and Population Health, University of Sheffield, Sheffield, UK; Sheffield Vascular Institute, Sheffield Teaching Hospitals NHS Foundation Trust, Sheffield, UK

## Abstract

**Background:**

There are disparities in the prevalence of peripheral arterial disease (PAD). This study examined socioeconomic, ethnic, and geographical variations in population-based revascularization rates for PAD and the associations between these rates and PAD-related major lower limb amputation.

**Methods:**

Hospital admissions in England (April 2008–March 2018) for revascularization for moderate and severe PAD, and for major lower limb amputation, were examined using a population-based study design and Poisson and logistic regression.

**Results:**

In the 10-year time span, there were 309 839 revascularization admissions (44% severe PAD, 56% moderate PAD; > 90% White ethnicity) and 38 601 major lower limb amputations from a population aged ≥ 25 years of 36 million. Overall, 39.9% of patients with amputation had previously had revascularization. Revascularization rates for moderate and severe PAD and amputation rates increased with increasing socioeconomic deprivation. The ratio of amputations to revascularizations also increased with increasing deprivation, and patients admitted for revascularization from more deprived areas had more severe PAD. Revascularization rates for patients of Black ethnicity were generally substantially lower than those for patients of White ethnicity, the only exception being for severe PAD in patients aged ≥ 65 years. The amputation rate was also lower but the ratio of amputations to revascularizations, and the percentage of revascularization admissions with severe PAD, were higher for patients of Black relative to White ethnicity. Revascularization and amputation rates were substantially lower, and the ratio of amputations to revascularizations was also lower, for patients of Asian relative to White ethnicity, but patients of Asian ethnicity admitted for revascularization had a marginally higher percentage of severe PAD. There was substantial variation in revascularization and amputation rates across Integrated Care Board areas, with high rates in the North and in parts of the Midlands and South West. There was also wide variation in the ratio of amputations to revascularizations.

**Conclusion:**

There were substantial socioeconomic, geographical and ethnic disparities in revascularization rates in England.

## Teaser

This study examined socioeconomic, ethnic and geographical variations in population-based revascularization rates for peripheral arterial disease (PAD) in England, and associations between these rates and PAD-related major lower limb amputation. The ratio of amputations to revascularizations increased with increasing socioeconomic deprivation, and admissions for revascularization from deprived areas had more severe PAD. There was substantial variation in revascularization and amputation rates across integrated care board areas, with high rates in the North and in parts of the Midlands and South West, but also wide variation nationally in the ratio of amputations to revascularizations.

## Introduction

The prevalence of peripheral arterial disease (PAD) affecting the lower limbs increases with increasing age, with prevalence estimates ranging from 4.3% in low- and middle-income countries and 3.5% in high-income countries for people aged 40–44 years to 12.0% to 21.2% respectively in people aged 80–84 years^1^.

There is a socioeconomic gradient in the prevalence of PAD, with prevalence increasing with increasing levels of socioeconomic deprivation^2,3^. There is also evidence of ethnic variation in the prevalence of PAD, with some studies reporting higher prevalence for Black ethnicity and lower prevalence for Asian ethnicity compared with White ethnicity^4–6^.

The severity of PAD ranges from asymptomatic disease through intermittent claudication to chronic limb-threatening ischaemia. Treatment options include surgical revascularization for more severe disease^7,8^. Recent evidence from England shows that revascularization rates are higher in areas with higher levels of socioeconomic deprivation^9^. Studies of ethnic variation in revascularization have largely focused on outcomes following revascularization, with few studies examining population-based ethnic variation in revascularization rates^10^.

In England, where the National Health Service (NHS) provides universal free access to revascularization, a previous study suggested that there is regional variation in revascularization rates^11^. There is also evidence of regional variation in PAD-related major lower limb amputation rates^12^.

The aims of this study were to examine socioeconomic, ethnic, and geographical variations in population-based revascularization rates for PAD in England, and to examine the associations between these rates and amputation.

## Methods

### Hospital Episode Statistics

NHS England supplied the Hospital Episode Statistics (HES) data for admitted patient care used in this study, which included data on day-case admissions. The data were for a 10-year period from April 2008 to March 2018 (data were provided in financial years, which run from 1 April to 31 March the following year). A pragmatic approach was used to classify revascularization for PAD into two categories, namely moderate and severe, because admission records did not contain diagnosis codes that adequately differentiated between intermittent claudication and chronic limb-threatening ischaemia.

The classification used existing definitions that had been developed through an iterative process where sample sets of records were classified by a clinical consensus group established as part of a previous vascular research programme funded by the National Institute for Health and Care Research (NIHR)^13^. The categories produced demonstrated clear differences in demographic, process and outcome measures that were consistent with distinct groups based on the severity of PAD^13^.

All revascularization surgery (open and endovascular) for PAD performed in an emergency (unplanned) admission was classified as severe. In addition, any elective (planned) admission for revascularization that included an International Classification of Diseases, Tenth Revision (ICD-10) code^14^ for leg ulcer or that included aortoiliac bypass, femorodistal bypass, or extra-anatomical bypass was classified as severe. The remaining elective admissions for revascularization were classified as moderate. If an admission contained more than one revascularization procedure, the admission was classified on the basis of the most severe procedure.

HES admission records included information on ethnicity codes under the variable ‘ethnos’. These ethnicity codes were grouped into six categories: White, Black, Asian, Mixed, Others, and Missing. Co-morbidities were identified using ICD-10 codes in the first 14 diagnosis fields in the admission record. Eight co-morbidity categories were considered (see Results), based on the Royal College of Surgeons Charlson score and opinions from the expert clinical advisory panel for this project^13,15^.

For examining associations with amputation rates, methods from previous work on amputations were used^12,16^. All admissions for patients who had a major lower limb amputation (above- or below-knee amputation) were identified and linked using a pseudonymized identifier, and the index admission was defined as the first admission with a major lower limb amputation. Traumatic and malignant amputations were excluded. The linked data included patient-level information on revascularization either in the amputation admission or in admissions within 1 year preceding the index admission.

Revascularization and amputation procedures were identified using OPCS procedure codes, the standard classification system used in the English NHS^17^. The admission data sets were restricted to age at admission of ≥ 25 years.

The OPCS and ICD-10 codes and methodology used to identify revascularizations and amputations have been provided in detail in the full report from the previous vascular research programme funded by the NIHR^13^.

### Geography, population, and socioeconomic deprivation

A Lower Layer Super Output Area (LSOA) of residence was provided for each admission in the HES data set. LSOAs are census-defined areas with an average population of approximately 1500 people^18^. Because there were changes to a small number of LSOAs between the 2001 and 2011 national population censuses, the analysis was limited to 31 672 of the 32 844 LSOAs in 2011 (96.4%) to ensure consistency over the study period.

Mid-year population estimates for LSOAs by age and sex from 2008 to 2017 were used to calculate revascularization and amputation rates. However, ethnicity-specific population data by age and sex at the LSOA level were only available for the 2011 census year. As a result, revascularization rate estimates by ethnicity were restricted to HES data from the 5-year period spanning 2011 (April 2009–March 2014) and calculated in broad age bands because of the more limited population data set.

Socioeconomic deprivation at the LSOA level was assessed using the Income Domain from the Index of Multiple Deprivation (IMD) 2010, a widely used national measure of deprivation in England^19^.

Geographical variation in revascularization rates was examined using the 42 integrated care board (ICB) areas covering England. ICBs are formally established statutory NHS organizations responsible for planning health services for their local population^20^, and LSOAs were assigned to ICBs using the corresponding look-up tables.

### Study design and statistical analysis

A population-based (ecological) study design was used. Poisson regression was used to examine population-based revascularization rates in relation to deprivation and ethnicity, and results are presented as adjusted rate ratios (RRs) with their 95% confidence intervals (c.i.). Data were very sparse at the LSOA level. Because of this, LSOAs were grouped into five categories using socioeconomic deprivation quintiles, and the median deprivation value within each category was used as a continuous variable in the statistical analyses. RRs were calculated as a trend across all quintile categories and are presented as the ratio (with 95% c.i.) for the most deprived category relative to the least deprived category.

Both revascularization rates and amputation rates could depend, to some extent, on the underlying prevalence of PAD. Therefore, when examining associations between revascularization and amputation, the ratio of amputations to revascularizations within socioeconomic or ethnic categories or ICBs was used to adjust for variation in underlying PAD prevalence. Logistic regression was used to analyse these ratios and to analyse data on previous revascularization in patients who underwent amputation.

## Results

In the 10-year time span analysed, there were 309 839 admissions for revascularization for PAD, of which 44% were for severe PAD and 56% for moderate PAD. The admissions were from a population aged ≥ 25 years of 36 million. The overall annual revascularization rate for PAD was 86 per 100 000 population.

Patients admitted for revascularization for severe PAD were marginally older, with a relatively lower proportion of men compared with admissions for revascularization for moderate PAD, although the majority of patients in both categories were men (*Table 1*).

**Table 1:** Characteristics of admissions for revascularization for moderate and severe PAD, with age on admission of ≥ 25 years, England, April 2008–March 2018

Characteristics	Revascularization for moderate PAD	Revascularization for severe PAD	Total
No. of admissions	174 014	135 825	309 839
Age (years), mean(s.d.)	68.4(10.9)	70.6(12.9)	69.4 (11.9)
**Sex (%)**			
Male	69.2	63.8	66.9
Female	30.8	36.2	33.1
Deprivation category*			
1 (most deprived)	39 705 (22.8%)	32 992 (24.3%)	72 697 (23.5%)
2	37 750 (21.7%)	30 003 (22.1%)	67 753 (21.9%)
3	35 756 (20.5%)	28 063 (20.7%)	63 819 (20.6%)
4	32 820 (18.9%)	24 712 (18.2%)	57 532 (18.6%)
5 (least deprived)	27 983 (16.1%)	20 055 (14.8%)	48 038 (15.5%)
Total	174 014 (100%)	135 825 (100%)	309 839 (100%)
Ethnicity*			
White	164 043 (94.3%)	126 900 (93.4%)	290 943 (93.9%)
Black	1821 (1.0%)	2685 (2.0%)	4506 (1.5%)
Asian	2970 (1.7%)	2701 (2.0%)	5671 (1.8%)
Mixed	435 (0.2%)	398 (0.3%)	833 (0.3%)
Others	1171 (0.7%)	1188 (0.9%)	2359 (0.8%)
Missing	3574 (2.1%)	1953 (1.4%)	5527 (1.8%)
Total	174 014 (100%)	135 825 (100%)	309 839 (100%)
Operation type*			
Aortoiliac bypass		15 261 (11.2%)	15 261 (4.9%)
Femorodistal bypass		9094 (6.7%)	9094 (2.9%)
Extra-anatomical bypass		9395 (6.9%)	9395 (3.0%)
Femoroproximal bypass	17 716 (10.2%)	17 063 (12.6%)	34 779 (11.2%)
Femoral endarterectomy	18 145 (10.4%)	11 645 (8.6%)	29 790 (9.6%)
Iliac angioplasty	43 651 (25.1%)	11 824 (8.7%)	55 475 (17.9%)
Femoral angioplasty	66 250 (38.1%)	42 693 (31.4%)	108 943 (35.2%)
Unspecified angioplasty	28 252 (16.2%)	18 850 (13.9%)	47 102 (15.2%)
Total	174 014 (100%)	135 825 (100%)	309 839 (100%)
**Co-morbidities†**			
Coronary artery disease	43 046 (24.7%)	39 509 (29.1%)	82 555 (26.6%)
Heart failure	5578 (3.2%)	14 921 (11.0%)	20 499 (6.6%)
Cerebrovascular disease	4603 (2.6%)	7674 (5.6%)	12 277 (4.0%)
COPD	27 645 (15.9%)	25 701 (18.9%)	53 346 (17.2%)
Diabetes	42 869 (24.6%)	48 082 (35.4%)	90 951 (29.4%)
Renal disease	13 491 (7.8%)	20 854 (15.4%)	34 345 (11.1%)
Cancer	4189 (2.4%)	7284 (5.4%)	11 473 (3.7%)
Moderate or severe liver disease	115 (0.1%)	483 (0.4%)	598 (0.2%)

Values are *n* (%) unless otherwise stated. *Percentages are for the column. †Percentage of presentations with co-morbidity. For moderate PAD, all operation types were elective admissions with no leg ulcers. For severe PAD, the operation types were as follows: elective and emergency admissions (aortoiliac bypass, femorodistal bypass, extra-anatomical bypass); and emergency admissions or elective admissions with leg ulcers (femoroproximal bypass, femoral endarterectomy, iliac angioplasty, femoral angioplasty, unspecified angioplasty). PAD, peripheral arterial disease; s.d., standard deviation; COPD, chronic obstructive pulmonary disease.

Patients admitted for revascularization for PAD from socioeconomically deprived areas tended to have more severe PAD. When examined within deprivation categories, the percentage of admissions for revascularization for PAD that were in the severe category was 45.4% of admissions in the most deprived category. This percentage decreased gradually with decreasing levels of deprivation to 41.7% in the least deprived category.

With regard to ethnicity, whilst the great majority (> 90%) of patients admitted for revascularization in both moderate and severe categories were of White ethnicity, a relatively higher proportion of patients in the severe PAD category were of Black and Asian ethnicity compared with patients in the moderate PAD category (2% *versus* 1% for Black ethnicity; 2% *versus* 1.7% for Asian ethnicity). When examined within ethnicity categories, 59.6% and 47.6% of patients admitted for revascularization were in the severe category for Black and Asian ethnicities, respectively, compared with 43.6% for White ethnicity.

With regard to type of operation, the main procedure for severe PAD was femoral angioplasty (31.4%), whereas the main procedures for moderate PAD were femoral angioplasty and iliac angioplasty (38.1% and 25.1%, respectively).

With regard to the prevalence of co-morbidities, there was a clear contrast between the severe PAD and moderate PAD categories. The prevalence of co-morbidities was higher in the severe PAD category for all eight co-morbidity categories.

There were 38 601 major lower limb amputations (index admissions) over the 10-year period analysed, giving an overall amputation rate of 10.7 per 100 000 population aged ≥ 25 years. Overall, 39.9% of patients with major amputation had previously had revascularization. Amputation rates in relation to socioeconomic deprivation, ethnicity, and geography have been analysed and presented in detail previously^12,16^. Here, the patterns of association between revascularization rates, amputation rates and the percentage of amputations with previous revascularization in relation to socioeconomic deprivation, ethnicity, and geography are presented.

### Socioeconomic deprivation and revascularization rates

**Fig. 1:**
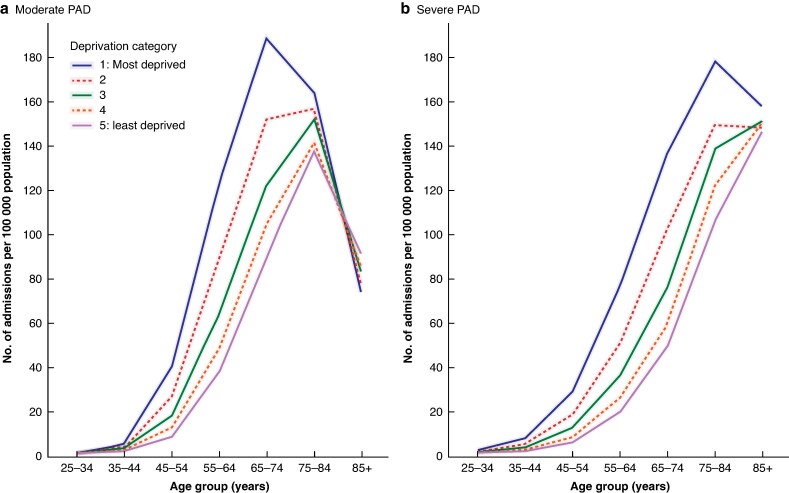
Average annual revascularization rates for moderate and severe PAD by age and socioeconomic deprivation, England, April 2008–March 2018 Revascularization rates for **a** moderate and **b** severe PAD. PAD, peripheral arterial disease.

Revascularization rates for moderate and severe PAD by socioeconomic deprivation and age are shown in *Fig. 1*. Revascularization rates increased with increasing levels of socioeconomic deprivation and the association was clearly seen for both the moderate and severe PAD categories. Revascularization rates increased with increasing age, reaching a peak in the 65–74- and 75–84-year age categories for moderate PAD, and the 75–84- and ≥ 85-year age categories for severe PAD.

In relative terms, the association between socioeconomic deprivation and revascularization rates was greatest in the 45–54-year age band in the moderate PAD category, with an RR adjusted for sex and year of 3.69 (95% c.i. 3.42 to 3.98) in the most relative to the least deprived category (*Table 2*). The strength of the association diminished with increasing age and, in fact, marginally reversed in the ≥ 85-year age band, with an adjusted RR of 0.83 (95% c.i. 0.79 to 0.89) in the most relative to least deprived category.

**Table 2:** Population-based revascularization rates for moderate and severe PAD by socioeconomic deprivation category (1–5) and age, and RRs (adjusted for sex and year) for the most deprived relative to the least deprived category, England, April 2008–March 2018

Age (years)	*N*	Revascularization rate per 100 000 population per year					RR*
		1 (most deprived)	2	3	4	5 (least deprived)	
**Revascularization for moderate PAD**							
25–34	892	1.4	1.3	1.3	1.4	1.1	1.07 (0.90, 1.28)
35–44	2537	5.8	4.1	3.3	2.5	2.2	2.47 (2.23, 2.72)
45–54	15 088	40.6	27.1	18.5	13.2	9.1	3.69 (3.42, 3.98)
55–64	41 408	123.6	89.9	64.4	48.5	37.8	2.95 (2.78, 3.15)
65–74	60 135	188.9	152.1	122.8	104.9	90.6	2.03 (1.93, 2.12)
75–84	44 009	164.1	157	152	141.7	138	1.22 (1.17, 1.26)
≥ 85	9945	73.9	77.3	83	85.3	91.2	0.83 (0.79, 0.89)
**Revascularization for severe PAD**							
25–34	1587	3.2	2.4	2	1.8	1.6	1.95 (1.72, 2.22)
35–44	3429	8.3	5.8	4.2	3.2	2.5	2.92 (2.67, 3.19)
45–54	10 637	29.2	19.1	13.2	8.7	6.4	3.82 (3.53, 4.14)
55–64	23 944	77.4	51.5	36.8	26.5	20.1	3.38 (3.17, 3.62)
65–74	38 099	137.4	103.2	76.3	59.4	49.5	2.66 (2.52, 2.80)
75–84	40 023	178.3	149.7	138.5	122.6	106.3	1.64 (1.57, 1.71)
≥ 85	18 106	157.9	148	151.7	150.7	147	1.07 (1.02, 1.13)

^*^Values in parentheses are 95% confidence intervals. N, number of patients; RR, rate ratio.

The association in relative terms for the severe PAD category between deprivation and revascularization rates also peaked in the 45–54-year age band, with an adjusted RR of 3.82 (95% c.i. 3.53 to 4.14) for the most deprived relative to least deprived category. The strength of the association diminished thereafter, but there was no reversal in association in the ≥ 85-year age band, with an adjusted RR of 1.07 (95% c.i. 1.02 to 1.13) in this age band.

### Associations between socioeconomic variation in revascularization and amputation

The patterns for major lower limb amputation rates and overall revascularization rates were similar, with both rates increasing with increasing levels of socioeconomic deprivation (*Table 3*).

**Table 3:** Socioeconomic deprivation and peripheral arterial disease-related revascularization rates, major lower limb amputation rates, ratio of amputations to revascularizations, and the percentage of amputations with previous revascularization, with corresponding RRs and ORs (relative to the least deprived category), for the population aged ≥ 25 years in England, April 2008–March 2018

	Socioeconomic deprivation				
	1 (Most deprived)	2	3	4	5 (Least deprived)
Revascularization rate (per 100 000 population per year)	107.7 (*n* = 72 697)	94.5 (*n* = 67 753)	86.4 (*n* = 63 819)	77.2 (*n* = 57 532)	66.3 (*n* = 48 038)
Revascularization RR*	1.62 (1.61, 1.64)	1.43 (1.41, 1.44)	1.30 (1.29, 1.32)	1.16 (1.15, 1.18)	1 (Reference)
Amputation rate (per 100 000 population per year)	15.6 (*n* = 10 530)	12.6 (*n* = 9015)	10.7 (*n* = 7900)	8.7 (*n* = 6453)	6.5 (*n* = 4703)
Amputation RR*	2.40 (2.32, 2.49)	1.94 (1.87, 2.01)	1.65 (1.59, 1.71)	1.33 (1.28, 1.39)	1 (Reference)
Ratio of amputations to revascularizations	0.14 (10 530 : 72 697)	0.13 (9015 : 67 753)	0.12 (7900 : 63 819)	0.11 (6453 : 57 532)	0.10 (4703 : 48 038)
OR for amputations to revascularizations*	1.48 (1.43, 1.53)	1.36 (1.31, 1.41)	1.26 (1.22, 1.31)	1.15 (1.10, 1.19)	1 (Reference)
% Amputations with previous revascularization	40.4 (4259 of 10 530)	39.2 (3538 of 9015)	39.6 (3132 of 7900)	40.0 (2583 of 6453)	40.5 (1904 of 4703)
OR for previous revascularization*	1.00 (0.93, 1.07)	0.95 (0.88, 1.02)	0.97 (0.90, 1.04)	0.98 (0.91, 1.06)	1 (Reference)

^*^Values in parentheses are 95% confidence intervals. RR, rate ratio; OR, odds ratio.

However, the ratio of amputations to revascularizations also increased with increasing levels of socioeconomic deprivation, rising from 0.10 in the least deprived category to 0.14 in the most deprived category.

In contrast, the percentage of patients with major amputation who had had previous revascularization was similar across deprivation categories.

### Ethnicity and revascularization rates

For patients of Black ethnicity, revascularization rates for moderate PAD were substantially lower relative to patients of White ethnicity (*Table 4*). In the 25–64-year age band, the RR adjusted for sex, year, and socioeconomic deprivation was 0.24 (95% c.i. 0.21 to 0.28), whereas in the ≥ 65-year age band it was 0.62 (95% c.i. 0.55 to 0.69).

**Table 4:** Population-based revascularization rates for moderate and severe PAD by ethnicity and age, and RRs adjusted for sex and year (relative to White ethnicity) before and after additional adjustment for socioeconomic deprivation, England, April 2009–March 2014

	Average annual revascularization rate per 100 000 population		RR*	
Black relative to White ethnicity				
Age (years)	Black ethnicity	White ethnicity	Adjusted for sex and year	Adjusted for sex, year, and deprivation
Revascularization for moderate PAD				
25–64	8.4	25.5	0.34 (0.30, 0.39)	0.24 (0.21, 0.28)
≥ 65	104.7	138.7	0.75 (0.69, 0.81)	0.62 (0.55, 0.69)
Revascularization for severe PAD				
25–64	9.0	15.5	0.59 (0.52, 0.68)	0.41 (0.37, 0.47)
≥ 65	155.5	112.1	1.38 (1.28, 1.48)	1.08 (0.99, 1.18)

Asian relative to White ethnicity				
Age (years)	Asian ethnicity	White ethnicity	Adjusted for sex and year	Adjusted for sex, year, and deprivation
Revascularization for moderate PAD				
25–64	6.2	25.5	0.24 (0.22, 0.27)	0.19 (0.17, 0.22)
≥ 65	71.7	138.7	0.50 (0.43, 0.58)	0.44 (0.39, 0.50)
Revascularization for severe PAD				
25–64	5.0	15.5	0.32 (0.29, 0.36)	0.25 (0.22, 0.29)
≥ 65	57.8	112.1	0.50 (0.45, 0.56)	0.43 (0.39, 0.48)

^*^Values in parentheses are 95% confidence intervals. PAD, peripheral arterial disease; RRs, rate ratios.

For severe PAD, in the 25–64-year age band, the revascularization rate for patients of Black ethnicity was also substantially lower relative to that for patients of White ethnicity, with an RR adjusted for sex, year, and socioeconomic deprivation of 0.41 (95% c.i. 0.37 to 0.47). However, in the ≥ 65-year age band, the unadjusted rate for patients of Black ethnicity was higher than that for patients of White ethnicity. The RR adjusted for sex and year was 1.38 (95% c.i. 1.28 to 1.48), but additional adjustment for socioeconomic deprivation largely accounted for the raised rate, with the RR coming down to 1.08 (95% c.i. 0.99 to 1.18).

The revascularization rates for patients of Asian ethnicity were all substantially lower than those for patients of White ethnicity. For moderate PAD, the RR for patients of Asian relative to White ethnicity, adjusted for sex, year, and socioeconomic deprivation, was 0.19 (95% c.i. 0.17 to 0.22) in the 25–64-year age band and 0.44 (95% c.i. 0.39 to 0.50) in the ≥ 65-year age band. For severe PAD, the adjusted RRs were 0.25 (95% c.i. 0.22 to 0.29) in the 25–64-year age band and 0.43 (95% c.i. 0.39 to 0.48) in the ≥ 65-year age band.

### Associations between ethnicity-related variations in revascularization and amputation

The overall revascularization rate for patients of Black ethnicity was approximately half that for patients of White ethnicity. The major lower limb amputation rate for patients of Black ethnicity was also lower, at 59% of that for patients of White ethnicity (*Table 5*). However, the ratio of amputations to revascularizations was higher for patients of Black ethnicity than for patients of White ethnicity (0.16 *versus* 0.13, respectively). The percentage of amputations with previous revascularization for patients of Black ethnicity was based on relatively small numbers and was broadly similar to that for patients of White ethnicity.

**Table 5:** Ethnicity and peripheral arterial disease-related revascularization rates, major lower limb amputation rates, ratio of amputations to revascularizations, and the percentage of amputations with previous revascularization, with corresponding RRs and ORs (relative to White ethnicity), in the population aged ≥ 25 years in England, April 2009–March 2013

	White ethnicity	Black ethnicity	Asian ethnicity
Revascularization rate (per 100 000 population per year)	95.5 (*n* = 149 804)	43.6 (*n* = 2239)	22.8 (*n* = 2673)
Revascularization RR*	1 (Reference)	0.46 (0.44, 0.48)	0.24 (0.23, 0.25)
Amputation rate (per 100 000 population per year)	11.9 (*n* = 18 735)	7.1 (*n* = 363)	2.4 (*n* = 283)
Amputation RR*	1 (Reference)	0.59 (0.53, 0.66)	0.20 (0.18, 0.23)
Ratio of amputations to revascularizations	0.13 (18 735 : 149 804)	0.16 (363 : 2239)	0.11 (283 : 2673)
OR for amputations to revascularizations*	1 (Reference)	1.30 (1.16, 1.45)	0.85 (0.75, 0.96)
% Amputations with previous revascularization	39.4 (7385 of 18 735)	41.6 (151 of 363)	35.3 (100 of 283)
OR for previous revascularization*	1 (Reference)	1.10 (0.89, 1.35)	0.84 (0.66, 1.07)

^*^Values in parentheses are 95% confidence intervals. RR, rate ratio; OR, odds ratio.

The overall revascularization rate for patients of Asian ethnicity was approximately one-quarter of that for patients of White ethnicity. The major lower limb amputation rate for patients of Asian ethnicity was also lower, at one-fifth of that for White ethnicity. The ratio of amputations to revascularizations was also lower for patients of Asian ethnicity than for patients of White ethnicity (0.11 *versus* 0.13, respectively). The percentage of amputations with previous revascularization for patients of Asian ethnicity was based on relatively small numbers and was broadly similar to that for patients of White ethnicity.

### Geographical variation in revascularization and amputation

**Fig. 2:**
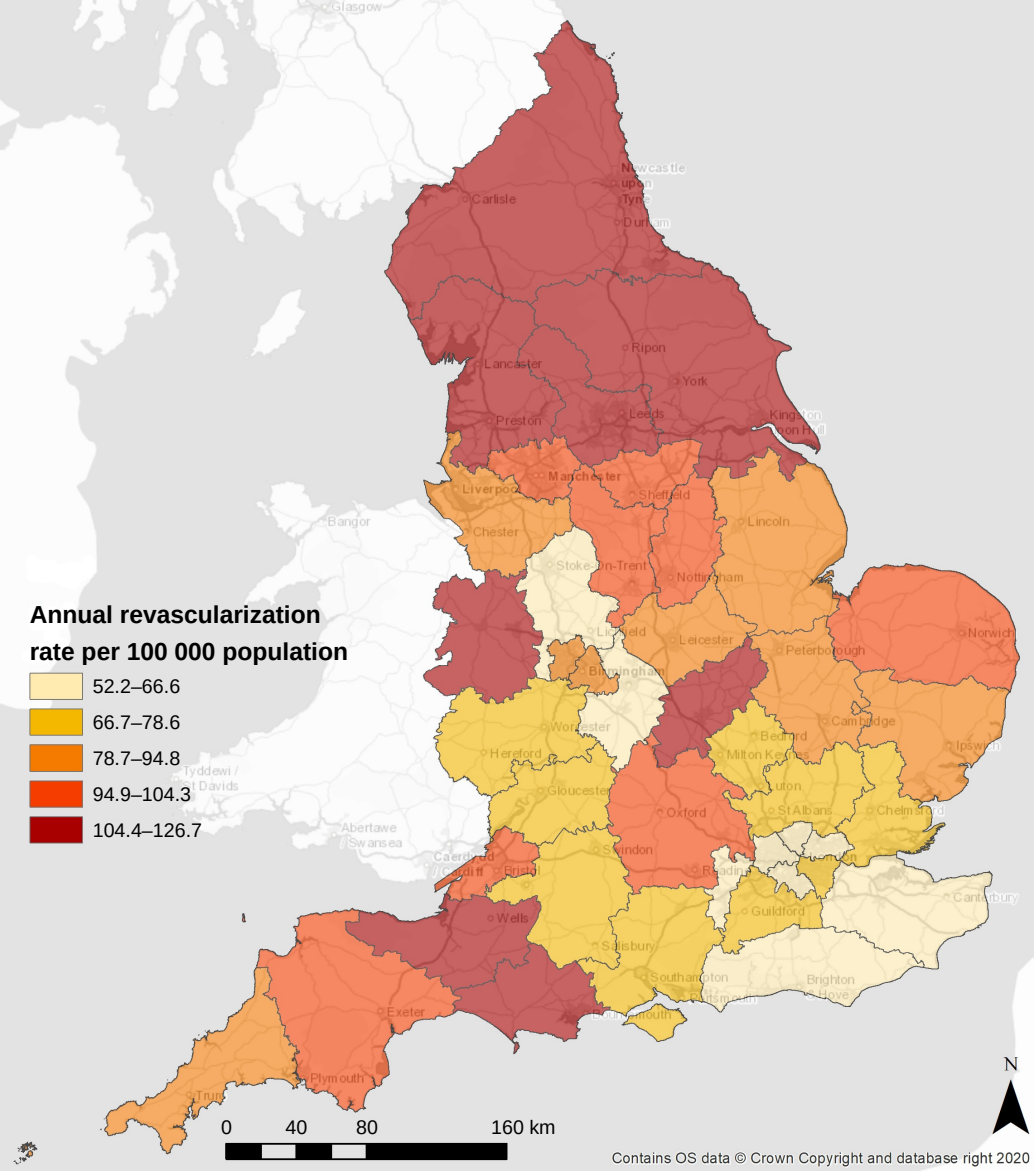
Average annual revascularization rates for peripheral arterial disease at the NHS Integrated Care Board level, England, April 2008–March 2018

There was a 2.4-fold variation in average annual revascularization rates across ICBs (*Table 6*), with revascularization rates ranging from 52.2 per 100 000 population in NHS North East London to 126.7 per 100 000 population in NHS Somerset (*Table 7*). In terms of geographical variation, there were high revascularization rates in the North of England, but also in parts of the Midlands and the South West (*Fig. 2*). (Although standardization for age and sex caused some changes, the overall geographical pattern observed using these standardized rates (*Fig. S1*) was broadly similar.)

**Table 6:** Descriptive statistics for NHS Integrated Care Board-level peripheral arterial disease-related revascularization rates, major lower limb amputation rates, ratio of amputations to revascularizations, and the percentage of amputations with previous revascularization for the population aged ≥ 25 years in England, April 2008–March 2018

	*N*	Mean(s.d.)	Median	Range
Average annual revascularization rate (per 100 000)	42	86.0(19.9)	89.9	52.2–126.7
Average annual major lower limb amputation rate (per 100 000)	42	10.6(2.5)	10.8	5.5–15.8
Ratio of amputations to revascularizations	42	0.13(0.03)	0.12	0.08–0.19
% Amputations with previous revascularization	42	39.8(3.6)	40.3	31.8–46.2

N, number of Integrated Care Boards; NHS, National Health Service; s.d., standard deviation.

**Table 7:** Peripheral arterial disease-related revascularization rates, major lower limb amputation rates, ratio of amputations to revascularizations, and the percentage of amputations with previous revascularization for NHS Integrated Care Boards for the population aged ≥ 25 years in England, April 2008–March 2018

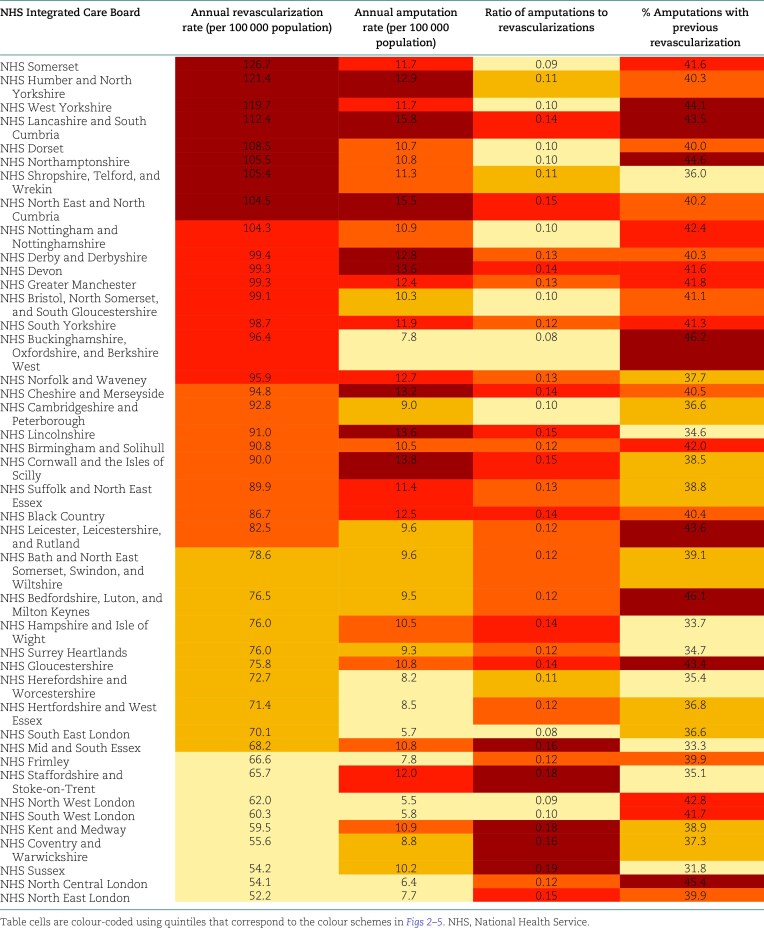

**Fig. 3:**
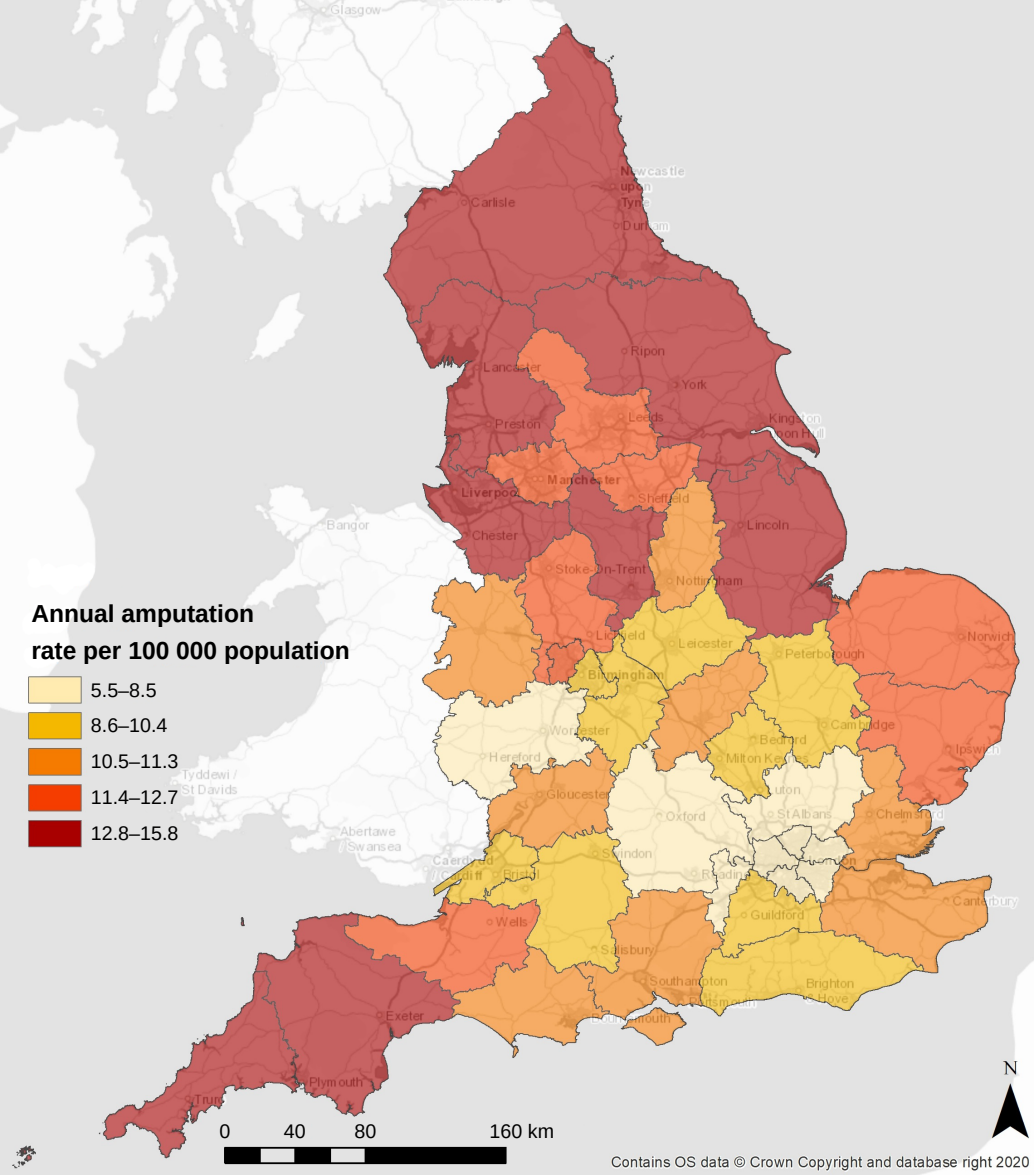
Average annual major lower limb amputation rates for peripheral arterial disease at the NHS Integrated Care Board level, England, April 2008–March 2018

There was a 2.9-fold variation in average annual major lower limb amputation rates across ICBs (*Table 6*), with amputation rates ranging from 5.5 per 100 000 population in NHS North West London to 15.8 per 100 000 population in NHS Lancashire and South Cumbria (*Table 7*). In terms of geographical variation, there were high rates of major lower limb amputation in the North of England, as well as in the northern parts of the Midlands and in the South West (*Fig. 3*). The overall pattern was generally broadly similar to revascularization rates.

**Fig. 4:**
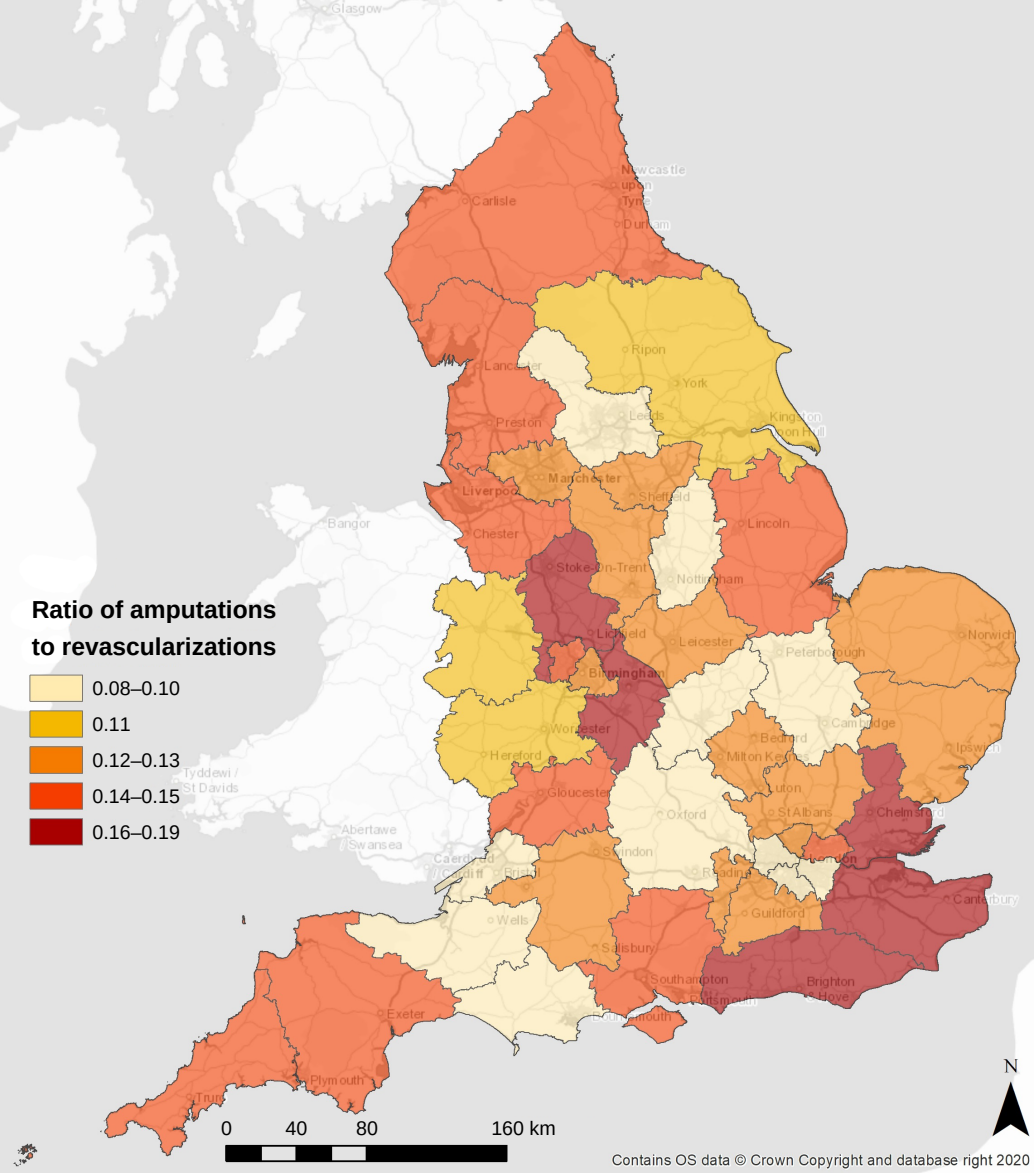
Ratio of major lower limb amputations to revascularizations for peripheral arterial disease at the NHS Integrated Care Board level, England, April 2008–March 2018)

There was a 2.4-fold variation in the ratio of amputations to revascularizations across ICBs (*Table 6*), with ratios ranging from 0.08 in NHS Buckinghamshire, Oxfordshire, and Berkshire West and in NHS South East London to 0.19 in NHS Sussex (*Table 7*). In terms of geographical variation, there was no clear geographical pattern and, in particular, little to suggest that the ratio of amputations to revascularizations was especially high in the North (*Fig. 4*).

**Fig. 5:**
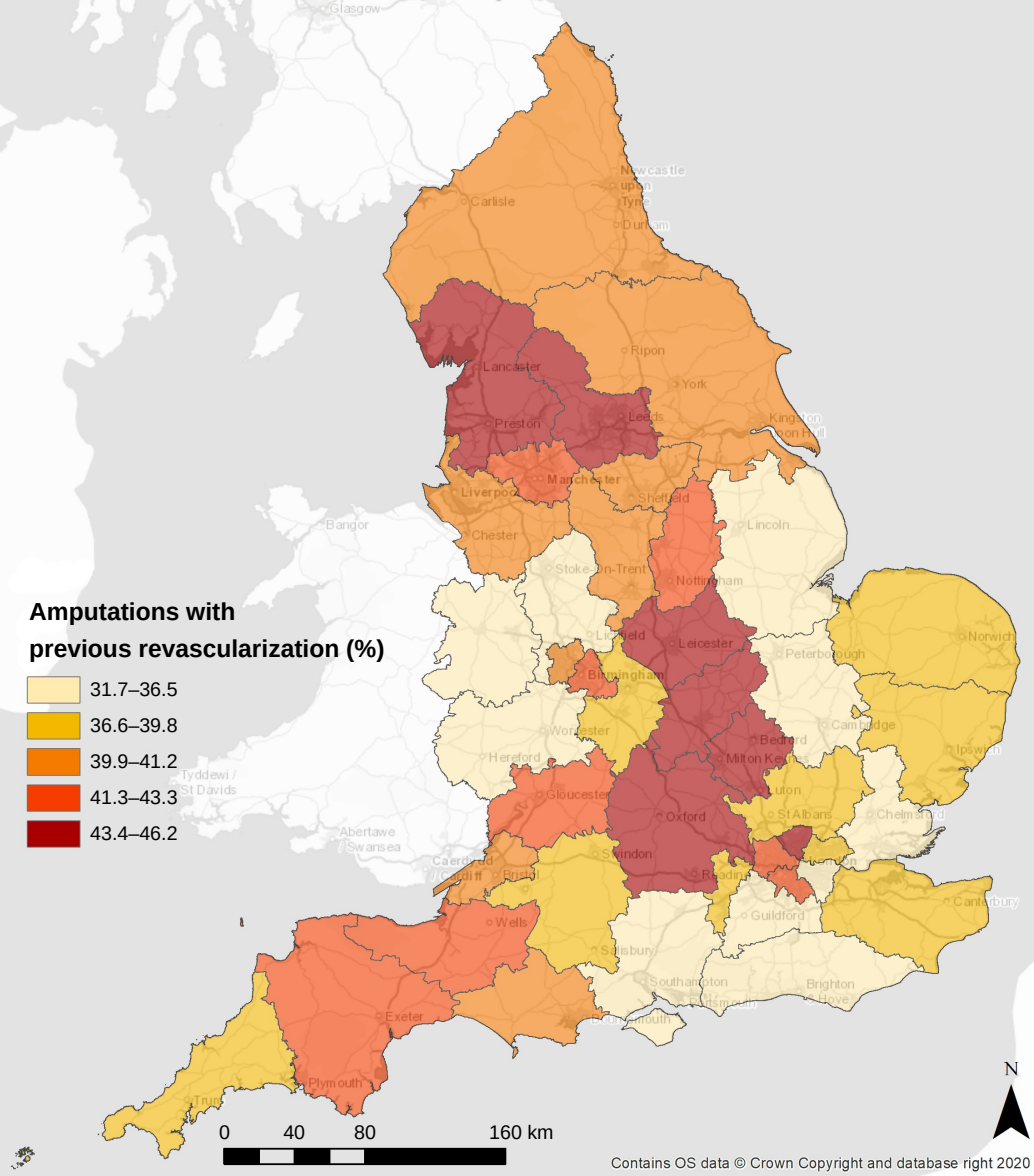
Percentage of major lower limb amputations with previous revascularization for peripheral arterial disease at the NHS Integrated Care Board level, England, April 2008–March 2018

There was a 1.5-fold variation in the percentage of amputations with previous revascularization across ICBs (*Table 6*), with percentages ranging from 31.8% in NHS Sussex to 46.2% in NHS Buckinghamshire, Oxfordshire, and Berkshire West (*Table 7*). In terms of geographical variation, there was no clear overall pattern and no indication that ICBs in the North were less likely to perform revascularization before amputation (*Fig. 5*).

## Discussion

Revascularization rates for PAD increased with increasing levels of socioeconomic deprivation, and this was apparent for both moderate and severe PAD across most of the adult age spectrum. Major lower limb amputation rates increased with increasing deprivation, but the ratio of amputations to revascularizations also increased with increasing levels of socioeconomic deprivation. In addition, admissions for revascularization from more deprived areas had more severe PAD. However, the percentage of patients with major amputation who had had a previous revascularization was similar across deprivation categories.

Revascularization rates for patients of Black ethnicity were generally substantially lower than those for patients of White ethnicity, with the only exception being the ≥ 65-year age group, where rates for severe PAD were similar after adjustment for deprivation. The major lower limb amputation rate for patients of Black ethnicity was also lower than that for patients of White ethnicity. However, the ratio of amputations to revascularizations was higher for patients of Black relative to White ethnicity. In addition, a higher percentage of admissions for revascularization in patients of Black ethnicity category was for severe PAD compared with patients of White ethnicity category. However, the percentage of amputations with previous revascularization for patients of Black and White ethnicity categories was broadly similar.

Revascularization rates and the major lower limb amputation rate were substantially lower for patients of Asian relative to White ethnicity. The ratio of amputations to revascularizations for patients of Asian ethnicity was also lower relative to patients of White ethnicity. However, a marginally higher percentage of admissions for revascularization in patients of Asian ethnicity category was for severe PAD compared with patients of White ethnicity category. The percentage of amputations with previous revascularization for patients of Asian and White ethnicity categories was broadly similar.

There was substantial variation in revascularization and amputation rates across ICBs, with high rates in the North of England, but also in parts of the Midlands and the South West. Although there was wide variation in the ratio of amputations to revascularizations, there was little to suggest that the ratio was especially high in the North. There was less variation in the percentage of amputations with previous revascularization and no indication that this percentage was lower in the North.

Although the higher revascularization rates in more socioeconomically disadvantaged areas have been reported on previously^9^, the present study clearly shows this pattern exists across most of the adult age spectrum. Hicks *et al*.^21^ examined the rate of revascularization in patients with intermittent claudication using data from the USA and found that patients living in lower income counties had higher rates of intervention for claudication. The present study found that patients living in more disadvantaged areas in England had higher revascularization rates for both moderate and severe PAD. However, the denominator was the general population and not specifically patients with intermittent claudication or chronic limb-threatening ischaemia. Other studies have also examined population-based revascularization rates for PAD in England^10,11,22^ and elsewhere, for example in Japan^23^ and Canada^24^, but these studies do not appear to have reported population-based rates by socioeconomic deprivation category.

Higher revascularization rates and higher major amputation rates associated with higher levels of socioeconomic deprivation observed in the present study are both likely to reflect the higher prevalence of PAD in more disadvantaged areas^2,3^. Nevertheless, the higher population-based ratio of amputations to revascularizations in disadvantaged areas needs further consideration. Possible explanations include patients living in disadvantaged areas being relatively more likely to have amputation than revascularization, as well as being more likely to have amputation after revascularization. In the USA, Mota *et al*.^25^ found that patients from disadvantaged areas were relatively more likely to have amputation than revascularization, whereas Heikkila *et al*.^26^ found that patients living in disadvantaged areas in England were more likely to have amputation after revascularization.

The present study found no association between socioeconomic deprivation and previous revascularization in patients who had undergone major lower limb amputation. This is consistent with the study by Ahmad *et al.*^11^, who found that among patients who underwent major lower limb amputation in England, there was no association between area-level deprivation and revascularization. In contrast, in a study of Medicare patients in the USA who had major amputation for chronic limb-threatening ischaemia, Ramadan *et al.*^27^ found that disadvantaged patients having major amputation were less likely to have had previous revascularization.

The present study found that patients from disadvantaged areas admitted for revascularization had more severe disease. This is consistent with the findings of studies by Chen *et al.*^28^ and Mota *et al.*^25^ in the USA, both of which also found that patients having revascularization for PAD had more severe disease if they were socioeconomically disadvantaged.

With regard to Black ethnicity, the present study found that revascularization rates were generally substantially lower for Black relative to White ethnicity. In order to interpret these population-based revascularization rates, knowledge of the underlying prevalence of PAD would be useful. However, there appear to be no large-scale studies of PAD incidence or prevalence for Black relative to White ethnicity in England. A small-scale study in a local area in England reported similar prevalence for Asian and Black ethnicities, but it was based on very small numbers and did not report on comparisons with White ethnicity^29^. In contrast, in the USA, an analysis using data from seven community-based studies found that PAD prevalence was higher for Black ethnicity than White ethnicity^4^. Another study^5^ using six cohorts in the USA also found similar results. However, a more recent cohort study from the USA that examined the incidence of PAD found that the unadjusted incidence was similar for Black and White ethnicities^30^.

Another way to interpret population-based revascularization rates is by comparison with major amputation rates. The present study found that both overall revascularization rates and amputation rates were lower for Black relative to White ethnicity. An earlier study using HES data from 2003 to 2009 for England found that patients of Black ethnicity was associated with lower revascularization rates but higher amputation rates when compared with patients of White ethnicity^10^, which would have raised concerns of disparity in the management of PAD. The findings from the present study suggest that the situation may have improved. However, the findings that the ratio of amputations to revascularizations was higher for Black relative to White ethnicity and that a higher percentage of admissions for revascularization in patients of Black ethnicity category were for severe PAD compared with admissions in patients of White ethnicity category suggest that concerns remain regarding disparity in the management of PAD for patients of Black ethnicity. In the USA, the cohort study that observed a similar incidence of PAD for Black and White ethnicities found that Black ethnicity was associated with a higher amputation risk^30^. Other studies from the USA found that patients of Black ethnicity having amputation for chronic limb-threatening ischaemia were less likely to have had arterial investigation or revascularization^27^, and, paradoxically, higher rates of revascularization for intermittent claudication^21^. In addition, a review^31^ based largely on studies from the USA found that patients of Black ethnicity were more likely to have severe PAD at presentation and higher amputation rates following revascularization.

The situation regarding Asian ethnicity and PAD prevalence is clearer. A systematic review found that Asian ethnicity was associated with a paradoxically lower prevalence of PAD compared with White ethnicity, even though the prevalence of both coronary heart disease and diabetes is higher for South Asian ethnicity^6^. That review found that the lower PAD prevalence was apparent in the general population, in people with coronary heart disease, and in people with diabetes^6^. The findings of the present study showing lower revascularization and amputation rates for patients of Asian ethnicity, along with an earlier study that reported similar findings^10^, are consistent with the lower PAD prevalence for Asian ethnicity.

The geographical variation at the ICB level has two main features: generally higher revascularization and amputation rates in the North of England and substantial variation across ICBs.

The overall general pattern of both higher revascularization rates and amputation rates in the North suggests that both these may be a consequence of higher underlying prevalence of PAD in the North. Higher rates of revascularization and amputation in the North have been observed previously by Ahmad *et al.*^11^ using HES data for an earlier period (2003–2009). There was no evidence from the present study of relative underutilization of revascularization in the North because the ratio of amputations to revascularization was not higher and the percentage of amputations with previous revascularization was not lower in the North.

The substantial variation in revascularization and amputation rates, the ratio of amputations to revascularizations, and, to a lesser extent, the percentage of amputations with previous revascularization across ICBs suggests significant issues in relation to local access to and quality of vascular healthcare, including at the primary care level. Possible factors include delays in diagnosis, inadequate medical management, inadequate access to smoking cessation services and supervised exercise programmes, inadequate provision of and access to specialist vascular services, and variations in local policies and practice.

A key strength of the HES data set is its comprehensive coverage, because all hospital admissions are routinely recorded within the NHS system. This high level of data completeness enables reliable calculation of population-based rates.

However, several study limitations should be acknowledged. The classification and recording of ethnicity within HES, as well as in population estimates by ethnic group, can be challenging. Ethnicity is generally self-reported and may vary due to its subjective nature, with individuals potentially identifying differently depending on context or interpretation of the available categories. In addition, there is potential for data entry errors. Discrepancies may also arise due to differences in data collection methods between HES and census-based population estimates.

Socioeconomic deprivation was measured at the area level using the IMD Income Domain and does not necessarily reflect the socioeconomic situation of all people within each area. Individual-level socioeconomic data are not routinely collected within HES. The use of IMD from a single reference year may have resulted in misclassification of some LSOAs, particularly towards the margins of the study period.

There is also the possibility of inaccuracies in the diagnostic and procedure codes recorded in HES, which may have varied over time and resulted in some misclassification of procedures and co-morbidities. In addition, the methodology used could have resulted in some misclassification between moderate and severe PAD. As an administrative data set, HES includes limited clinical detail. Furthermore, LSOA-level population estimates are subject to uncertainty and may not precisely represent actual population counts.

From a policy perspective, socioeconomic disparities in PAD need to be clearly recognized in vascular policy documentation in order to influence clinical practice. Neither the 2016 Best Practice Clinical Care Pathway for major amputation surgery nor the 2022 update of the Best Practice Clinical Care Pathway for PAD for the UK acknowledge socioeconomic disparities^32,33^. The National Vascular Registry Healthcare Improvement Strategy for the UK, published in 2023, is relevant to a wider audience but also does not mention socioeconomic disparities in PAD^34^, and there is no mention of socioeconomic disparities in the National Vascular Registry 2024 State of the Nation Report^35^. In addition, the UK All-Party Parliamentary Group on Vascular and Venous Disease 2019 report entitled Saving Limbs, Saving Lives also does not highlight socioeconomic disparities in PAD^36^. However, the report does acknowledge that disparities exist in relation to Black ethnicity^36^.

In addition, in terms of implications, the key new findings relate to the substantial variation in the ratio of amputation to revascularization across ICBs. This indicates that further investigation is required to understand the reasons for this variation in order to improve access to vascular healthcare and the quality of that care. Potential reasons are that the distribution of disease or co-morbidities makes some groups less suitable for revascularization; that some groups have delayed presentation or referral, making revascularization less viable; or that there are biases, patient preferences, or local variations in the system that make it less likely for some groups to be considered for revascularization.

Improving care for leg ulcers has been identified as a key issue in recent reports from the UK All-Party Parliamentary Group on Vascular and Venous Disease^37,38^. The observed regional variation in amputation and revascularization rates supports the need for improved multidisciplinary working with integrated vascular, podiatry, and nursing community pathways to ensure early intervention and consistent care quality. Issues relating to workforce and training investment, including in district nursing and wound care services, also need to be addressed. In addition, improved community data capture to complement hospital data sets to better understand demand for services would be useful, with national standards for data linkage across care settings to monitor performance and outcomes.

## Supplementary Material

zrag081_Supplementary_Data

## Data Availability

The HES data used in this project may be obtained from NHS England.
