# Supplementary material for: Socioeconomic, ethnic and geographical disparities in revascularization rates for peripheral arterial disease in England
Source: BJS Open. 2026 Aug 5;10(4):zrag081. doi: 10.1093/bjsopen/zrag081 (PMC13439130; doi:10.1093/bjsopen/zrag081)
Supplement: Supplementary file 1 [file zrag081_supplementary_data.docx]

**Title: Socioeconomic, ethnic and geographical disparities in revascularisation rates for peripheral arterial disease in England**

Authors

Ravi Maheswaran^1^

Thaison Tong^1^

Jonathan Michaels^1^

Paul Brindley^2^

Stephen Walters^1^

Shah Nawaz^3^

^1^ School of Medicine and Population Health, University of Sheffield, United Kingdom

^2^ School of Architecture and Landscape, University of Sheffield, United Kingdom

^3^ Sheffield Vascular Institute, Sheffield Teaching Hospitals NHS Foundation Trust, United Kingdom

**Corresponding author.** Professor Ravi Maheswaran, Public Health, School of Medicine and Population Health, Regent Court, 30 Regent Street, Sheffield S1 4DA, United Kingdom **ORCID ID** 0000-0002-3899-4421

**Supplementary Materials - Index**

| **Supplementary Figure** |  |
| --- | --- |
| Figure 1. Age and sex standardized average annual revascularisation rates for PAD at the NHS Integrated Care Board level; England (April 2008 to March 2018). | *page 2* |
|  |  |
|  |  |
|  |  |

**Supplementary Figure**

**Figure 1. Age and sex standardized average annual revascularisation rates for PAD at the NHS Integrated Care Board level; England (April 2008 to March 2018).**
